# Mineral elements and adiposity-related consequences in adolescents with intellectual disabilities

**DOI:** 10.1186/s12860-023-00490-5

**Published:** 2023-09-20

**Authors:** Ahmad H. Alghadir, Sami A. Gabr, Amir Iqbal

**Affiliations:** Department of Rehabilitation Sciences, College of Applied Medical Sciences, King Saud University, P.O. Box 10219, Riyadh, 11433 Saudi Arabia

**Keywords:** Oxidative stress, Mineral elements, Adipokines, Intellectual disability, Adolescence

## Abstract

**Background:**

Patients with intellectual disabilities are shown to have a limited capacity for cooperation, communication,and other biological consequences, which significantly require a specialized interest in healthcare professionals worldwide.

**Aim:**

In this respect, the present study was designed to evaluate the levels mineral elements, and their correlation with oxidative stress markers and adiposity markers; leptin (L), adiponectin (A), and L/A ratio in adolescents with intellectual disabilities.

**Methods:**

A total of 350 schoolchildren aged (12–18 years) were randomly invited to participate in this prospective, observational study. Only 300 participants agreed to participate in this study. According to Intelligence quotients scores (IQ) measured by WISC-III, the participants were classified into two groups; the healthy control group (no = 180; IQ = 90–114); and the moderate intellectual disability (MID) group (no = 120; IQ = 35–49). Adiposity markers; body mass index (BMI), waist-to-hip ratio (WHR), waist-to-height ratio (WHtR), physical activity scores, adipokines biomarkers; leptin, adiponectin, L/A ratio, oxidative stress, and plasma mineral elements were evaluated by prevalidated questionnaires, inductively coupled plasma-mass spectrometry (ICP-MS), colorimetric, and immunoassay techniques.

**Results:**

Intellectual disability of moderate type was reported in 40% of the studied populations most of them are men aged 12–18 years (66.6% for men vs. 33.3 for females). Obesity was shown to be associated with the degree of intellectual disability of the students. There was a significant (*P* = 0.001) increase in the BMI, WHR, and WHtR scores as obesity markers with poor physical activity (*P* = 0.01) in students with poor disability compared to healthy controls (HC). The levels of leptin (*P* = 0.001), adiponectin (*P* = 0.01), and L/A ratio (*P* = 0.01) as adiposity biomarkers were significantly increased in students with MID compared to healthy controls. Also, oxidative stress measured by malondialdehyde (MDA) (*P* = 0.01) and total antioxidant capacity (TAC) (*P* = 0.01) were significantly increased in students with MID compared to healthy control subjects. In addition, mineral elements were shown to be linked with intellectual disability. The data showed that the levels of Fe, Mn, Zn, Hg, Pb, Ca, Cr, Mg, and Ni significantly (*P* = 0.001) increased, and the levels of Al, Na, K, Cu, and Zn/Cu ratio significantly (*P* = 0.001) decreased in subjects with MID compared to healthy controls. Correlation analysis concluded that changes in mineral elements significantly correlated with adiposity markers, oxidative stress, and the scores of intellectual disability (WISC III-IQ score).

**Conclusion:**

The intellectual disability of moderate type (MID) was associated with abnormal changes in the levels of essential mineral elements and adipokines and increased levels of cellular oxidative stress. Thus, evaluating plasma mineral elements and adipokines levels could be a potential diagnostic parameter for diagnosing MID.

## Introduction

Obesity is considered one of the most problematic issues of health worldwide. It affects all populations at different ages and socio-economic levels. Most studies reveal the contribution of obesity to human diseases, including diabetes mellitus [1, 2]. It becomes significantly associated with food containing high calories and poor physical activity. Several research studies linked obesity with economic, social, and lifestyle changes that lead to major public health problems [2–4]. In childhood, obesity is shown to be linked with several genetic and environmental factors. Lifestyle, social culture, and genealogy play a potential role in the spreading rates of obesity worldwide [4–8].

On biochemical and cellular levels, obesity is controlled by some proteins expressed from adipocytes, especially leptin. Leptin is a neurohormone (16 kDa) expressed by adipocytic cells and predominantly released into blood circulations. Physiologically, the release of leptin acts as a signal to the brain to control the storage of energy by the human body [9]. Thus, it was reported that leptin significantly controls food intake by inhibiting the release of Neuropeptide Y (NPY) through its hypothalamic receptors, reducing food intake with increased body thermogenesis [9].

Essential menirals and trace elements are cofactors essential for several cellular processes in human bodies. It plays potential roles in normal and diseased cells as regulatory, immunologic, or antioxidant factors, particularly as cofactors or essential components in the structures of cellular enzymes [10]. In obesity, trace elements are shown to be associated with the severity of the disease and its associated complications, such as peroxidation, inflammation, and metabolic disturbances [11, 12].

Previously, the deficiency in the levels of cellular micronutrients was reported to be significantly associated with fat deposition and chronic inflammation [13–15]. In addition, low levels of iron, zinc, and a deficiency in the levels of essential vitamins (A, E, and C) were estimated in children and adolescents with obesity compared to non obese controls of the same age [16–19]*.* These micronutrients, especially vitamins (A, E, and C), are essential for inhibiting or suppressing leptin expression [13, 20–22].

Several research studies reported a significant association between obesity levels and youth with intellectual and developmental disabilities. Obesity was significantly reported in children or adolescents with intellectual disabilities (ID). It was nearly twice the prevalence for those without ID (28.9% vs. 15.5%) [23, 24]. This may be related to poor physical activity and longer time spent sitting in front of screen-based media [25–30]. In childhood and adolescence, cognitive, behavioral, and neuropsychological defects; particularly ID, showed to be associated respectively with exposure to heavy metals such as arsenic (As), cadmium (Cd), manganese (Mn), mercury (Hg), and Lead (Pb) [31–33]. Reduced IQ and cognitive functions, learning difficulties, and impaired growth were reported in children with Pb blood levels above 10 μg·dL − 1 [34–37]. The pathophysiology of metal intoxication and producing intellectual or developmental defects may proceed with cellular free radical oxidative stress mechanisms [38, 39]. Higher malondialdehyde (MDA) and lower total antioxidant capacity (TAC) were reported as an indicator of cell membrane injury [38, 39]. In this respect, the present study was designed to evaluate the levels of mineral elements, and their correlation with oxidative stress markers and adiposity markers; leptin (L), adiponectin (A), and L/A ratio in adolescents with intellectual disabilities.

## Materials and methods

### Subjects

A total of 350 Saudi school students aged (12–18 years) attending various schools in Riyadh were randomly invited to participate in this study. Firstly, the school administration was notified about the need and importance of the study. Once necessary permission was obtained, they connected us with the students and their parents. Only 300 participants agreed to participate in this study. None of the selected participants have any physical disabilities, genetic disorders, or acute infections or received medical therapy for ID or obesity that had affected the data. Based on the intelligence quotients (IQ), the participants were classified into two groups; the normal healthy group (no = 180; IQ = 90–114); and the moderate ID group (no = 120; IQ = 35–49). Whole blood samples were collected from all participants and centrifuged ( 1 min at 1400 rpm), and the resulting plasma samples were kept frozen at—20°C until reused. Demographic and clinical data of the participants are in Table 1.

**Table 1:** Baseline of clinical and laboratory characteristics of the study groups. Healthy control (HC) and adolescents subjects suffering from moderate intellectual disabilities (MID) (*n* = 300; mean ± SD)

Parameters	HC (*n* = 180; 60%) (IQ = 85–114)	MID (*n* = 120; 40%) (IQ = 40–54)	*P*-value
Age in years	14.86 ± 2.5	14.9 ± 1.5	0.123
Genders (B/G)	120/60	80/40	0.13
BMI (kg/m2)	18.6 ± 2.3	32.8 ± 6.3	0.001
Waist (cm)	79.3 ± 5.1	116.3 ± 8.3	0.001
Hips (cm)	92.5 ± 2.6	78.9 ± 11.8	0.001
WHR	0.79 ± 0.029	1.47 ± 0.16	0.001
WHtR	0.46 ± 0.05	0.89 ± 0.09	0.001
Physical activity (PA):			0.01
VO_2_ max (ml/kg*min)	32.6 ± 4.31	21.3 ± 2.1	
BMR (kcal/day)	3.6 ± 2.5	1.36 ± 1.4	
TEE (kcal/day)	6.7 ± 5.3	2.9 ± 1.6	
PA scores	4.9 ± 3.1	1.9 ± 1.25	
WISC- IQ test scores	93.8 ± 2.6	39.2 ± 3.1	0.001

Values are expressed as mean ± SD; Kruskal–Wallis one-way ANOVA and post-hoc (Tukey HSD) test were used to compare the mean values of the studied variables. Variables were considered significantly different at *P* < 0.05

*Abbreviation*: *HC* Healthy control, *BMI* Body mass index, *WHR* Waist to hip ratio, *WHtR* Waist to height ratio, *PA* Physical activity, *VO*_*2*_* max* maximal oxygen uptake, *BMR* Basal metabolic rate (kcal/day), *TEE* Total energy expenditure (kcal/day), *WISC- IQ* Wechsler Intelligence Scale test

### Ethical considerations

The current protocol was prepared according to the ethical guidelines of the 1975 Declaration of Helsinki and finally reviewed and approved by the ethics sub-committee of King Saud University, Kingdom of Saudi Arabia, under file number ID: RRC-2015–089. All participating schoolchildren were informed of the steps and all protocol details. The participants’ parents were assigned to return written informed consent before data collection.

### Intelligence assessment

The participants’ intelligence quotients (IQ) were evaluated using a pre-validated Wechsler Intelligence Scale for Children (WISC-III), as previously reported [40, 41]. The results of IQ measured by WISC-III are categorized into seven scores; Mild intellectual disability (IQ 55–69), Moderate intellectual disability (IQ 40–54), below normal (IQ 70–84), normal (IQ 85–114), Above normal (IQ 115–129), Gifted (IQ 130–144), and Highly Gifted (IQ 145–160). In this study, IQ measurements of the participants were in the range of normal (IQ = 85–114; *n* = 180) and moderate (IQ = 40–54, *n* = 120), respectively.

### Anthropometric measurements

All participants’ height and weight were estimated using standardized procedures such as a tape measure and calibrated Salter Electronic Scales (Digital Pearson Scale; ADAM Equipment Inc., Columbia, MD, USA), respectively. Validated universal cutoff values [42, 43] were used to calculate adiposity parameters, such as BMI and Waist-to-height ratio (WHtR), respectively.

### Assessment of adiposity markers

Adiponectin and leptin levels as adiposity biomarkers were estimated in all participants' plasma samples using a specific ELISA kit (R&D Systems®, Minneapolis, USA). All samples were estimated in duplicate according to the manufacturer’s instructions to avoid inter-assay variation, as previously reported [44]. In contrast, the detection limits for adiponectin and leptin were 5 pg/mL, respectively [44].

### Assessment of essential mineral elements concentrations

In this experiment, plasma samples of all participants were subjected to estimate mineral elements concentrations by using a Thermo Fisher Scientific (Waltham, MA, USA) iCAP— Q instrument, equipped with standard components and accessories: a MicroMist™ nebulizer (Glass Expansion, Port Melbourne, Australia) as previously reported [45]. This method used multi-element standard solutions (Plasma CAL, SCP Science, Baie D’Urfé, Canada) to prepare calibration standards. In addition, an iso standard solution (Madrid, Spain) was used to prepare the internal standard solution. Ten replicate measurements of the blank solution (2% v/v HNO3) were performed to calculate the limits of detection (LoD) as previously reported [45].

### Assessment of oxidative stress

As a quantitative measure of lipid peroxidation, Malondialdehyde was estimated in the plasma samples using high-performance liquid chromatography, as mentioned previously [46–48]. In addition, a total antioxidant capacity (TAC), a measure of oxidative stress, was estimated in the plasma samples using a colorimetric assay kit (K274-100; BioVision, Milpitas, CA, USA). The antioxidant activity was measured as a function of Trolox concentration at a wavelength of (λ; 570 nm) as previously reported [47, 48].

### Assessment of physical activity

Physical fitness score is measured as maximum oxygen uptake (VO2 max) and total energy expenditure (TEE), as previously reported [29, 47–49]. Total energy expenditure (TEE) was evaluated by calculating basal metabolic rates (BMR) from body mass, height, age, sex, and type of physical activity of all participants using a pre-validated equation as previously reported [29, 47, 48].

### Statistical analysis

In this study, the statistical software SPSS version 18 was used. The results obtained were expressed as Mean, and standard deviation among groups, Kruskal–Wallis one-way ANOVA and post-hoc (Tukey HSD) test were used to compare the mean values of the studied variables [45]. Additionally, post hoc pairwise multiple comparisons using Bonferroni correction and the one-way analysis of covariance were performed to evaluate significant differences in trace elements hair contents between the study groups. The relationship between various study parameters was performed in steps by Spearman rank correlation analysis. The data obtained were considered significant at *P* < 0.05 [45].

## Results

The clinical and baseline characteristics of 300 adolescents with a mean range of age 14.9 ± 1.5 years who participated in this prospective study are shown in Table 1.

**Fig. 1:**
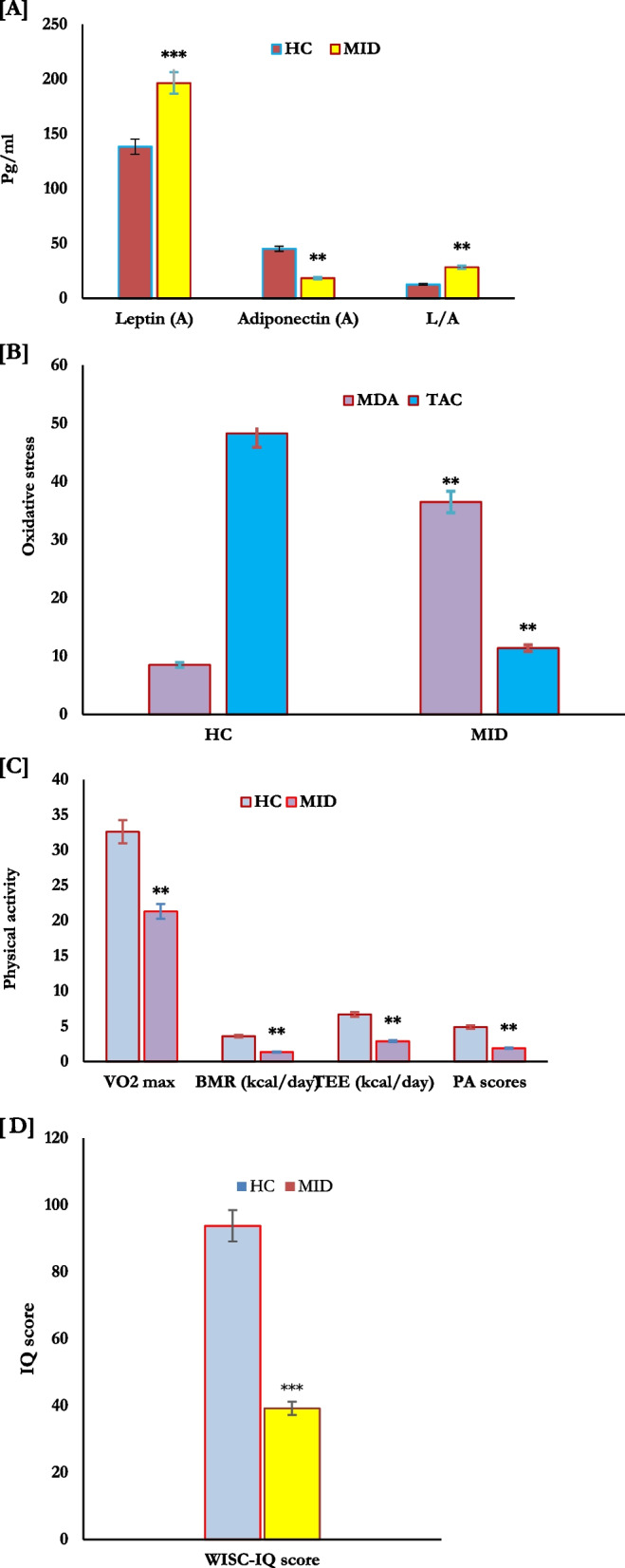
Plasma levels of adiponectin, leptin (L), and L/A ratio (pg/mL) (**A**), MDA and TAC (**B**), along with physical activity (**C**), and IQ-score (**D**) in HC (*n* = 180) and MID (*n* = 120). **p < 0.01 (KruskaleWalis, Dunn’s post hoc test). ***p < 0.001 (KruskaleWalis, Dunn’s post hoc test). HC: healthy controls, MID: moderate intellectual disability; IQ: Intelligence quotients; MDA: malondialdehyde, TAC: total antioxidant capacity

In this study, intellectual disability of moderate type (MID; WISC-IR score:39.2 ± 3.1) was reported in 40% of the study population, most of whom are men (66.6% for men vs. 33.3 for females) (Table 1). Compared to healthy control subjects, adiposity markers; BMI, waist, hips, WHR, and WHtR significantly increased (*P* = 0.001) in adolescents with MID (Table 1). In addition, physical activity scores measured in terms of VO2 max, BMR, and TEE significantly decreased (*P* = 0.01) in adolescents with (MID) compared to those of healthy controls (HC), as shown in Table 1 and Fig. 1. Also, IQ-score was lower in adolescents with MID compared to healthy controls, as shown in Table 1 and Fig. 1.

In this study, plasma mineral elements were significantly estimated in all participants (Table 2). Adolescents with MID showed a significant increase in the levels of Fe, Mn, Zn, Hg, Pb, Ca, Cr, Mg, and Ni, and they significantly decreased in Al, Na, K, Cu, and Zn/Cu ratio levels compared to healthy controls (Table 2).

**Table 2:** Mineral elements levels (µg/g) in plasma samples of the study participants, reported as mean ± SD and (range) according to the intellectual disability status measured by the Wechsler Intelligence Scale test. (HC = healthy control; MID = moderate intellectual disabilities

Element (µg/g)	HC (*n* = 180; 60%) (IQ = 85–114)	MID (*n* = 120; 40%) ( IQ = 40–54)	*P*-value
Elemental decrease (⬇)			
Al	4.5 ± 2.8	2.9 ± 1.8	0.001
Na	31.7 ± 4.7	18.2 ± 2.8	0.001
K	6.8 ± 4.6	4.7 ± 2.5	0.001
Zn/Cu ratio	0.69 ± 0.11	0.41 ± 0.18	0.001
Cu	116.3 ± 8.3	89.3 ± 12.8	0.001
Elemental increase (⬆)			
Zn	25.7 ± 3.1	36.5 ± 9.7	0.001
Fe	3.75 ± 2.8	8.7 ± 4.3	0.001
Hg	0.85 ± 0.89	1.7 ± 0.42	0.001
Pb	0.21 ± 0.12	0.42 ± 0.13	0.001
Ca	6.2 ± 1.5	8.7 ± 1.3	0.001
Cr	22.3 ± 4.6	31.1 ± 2.4	0.001
Mg	1.5 ± 1.1	2.9 ± 0.86	0.001
Ni	2.7 ± 0.25	4.2 ± 1.5	0.001
Mn	0.18 ± 0.120	0.95 ± 1.14	0.001

Data expressed as mean ± SD. Post hoc analysis using the Bonferroni method; The data obtained were deemed significant at *P* < 0.05 (HC vs. Moderate ID)

The physiological changes in the plasma levels of mineral elements correlated positively with the WISC-IQ score, estimating the potential role of these elements in the pathogenesis of intellectual disability among younger ages with MID (Table 3). Moreover, the results showed that the increase in the levels of Fe, Mn, Zn, Hg, Pb, Ca, Cr, Mg, and Ni, and the decrease in the levels Al, Na, K, Cu, and Zn/Cu ratiocorrelated positively with the cellular oxidative stress parameters; MDA, TAC, and negatively with adiposity parameters; BMI, WHR, and WHtR as shown in (Table 4).

**Table 3:** Correlation between plasma mineral elements with WISC-IQ score as a measure of ID in healthy control (HC) and adolescents with MID

Element (µg/g)	HC (*n* = 180; 60%) (IQ = 85–114)		MID (*n* = 120; 40%) ( IQ = 40–54)	
	R	P	R	P
Elemental decrease (⬇)				
Al	0.012	0.01	0.015	0.05
Na	0.015	0.05	0.025	0.05
K	0.036	0.05	0.039	0.05
Zn/Cu ratio	0.056	0.01	0.058	0.01
Cu	0.035	0.01	0.49	0.02
Elemental increase (⬆)				
Zn	0.4051	0.001	0.057	0.001
Fe	0.038	0.006	0.046	0.008
Hg	0.035	0.01	0.037	0.05
Pb	0.036	0.01	0.048	0.02
Ca	0.038	0.001	0.042	0.002
Cr	0.012	0.001	0.048	0.003
Mg	0.024	0.001	0.052	0.003
Ni	0.021	0.001	0.058	0.002
Mn	0.035	0.001	0.065	0.001

**Table 4:** Correlation between plasma mineral elements and adiposity parameters, WISC- IQ score,oxidative stress, and gender in adolescents with MID

Variables	Plasma Trace elements^c^			
	Elements with Increased values^a^		Elements with Decreased values^b^	
	R	P	R	P
Gender (M/F)	0.0125	0.12	0.034	0.18
WISC- IQ score	0.124	0.001	0.068	0.001
Adiposity paramters (BMI, WHR, WHtR)	-0.524	0.001	-0.089	0.001
Oxidative stress (MDA, TAC)	0.452	0.001	0.256	0.001

^a^{ Fe, Mn, Zn, Hg, Pb, Ca, Cr, Mg, Ni}; ^b^{ Al, Na, K, Cu, and Zn/Cu ratio}; ^c^Data are R (spearman)

However, increased or decreased levels of mineral elements showed no statistical significance with gender effect (Table 4). The increment of Fe, Mn, Zn, Hg, Pb, Ca, Cr, Mg, Ni, and decrement in the levels of Al, Na, K, Cu, and Zn/Cu ratio showed no significant effect with gender in subjects with ID (Table 4).

Also, leptin, adiponectin, and L/A ratio as adipokines biomarkers were estimated in all studied populations. Higher plasma levels of leptin and L/A ratio and lower adiponectin concentrations were reported in adolescents with MID (*P* = 0.001) compared with healthy controls (Fig. 1). In addition, MDA and TAC as parameters of oxidative stress were significantly evaluated in this study. The levels of MDA significantly increased, and TAC significantly decreased in adolescents with MID (*P* = 0.001) compared to healthy controls (Fig. 1).

In subjects with MID, the correlation between serum levels of adipokines and plasma mineral elements and clinically studied adiposity variables are shown in (Table 4). Leptin, adiponectin, and L/A as adiposity markers correlated negatively with BMI, WHtR, PA scores, and TAC and positively with gender, WISC-IQ score, MDA, and plasma trace elements (Table 5).

**Table 5:** Correlation between adipokines biomarkers with plasma mineral elements and clinically studied variables of adiposity in adolescents with MID

Variables	Adipokines (pg/ml) as markers of adiposity					
	Leptin (L)		Adiponectin (A)		L/A ratio	
	R	P	R	P	R	P
BMI	-0.215	0.01	-0.238	0.01	-0.324	0.001
WHR	-0.258	0.01	-0.342	0.01	-0.365	0.001
WHtR	-0.235	0.01	-0.369	0.05	-0.342	0.01
PA score	-0.251	0.05	-0.392	0.01	-0.259	0.001
Gender	0.328	0.01	0.393	0.01	0.254	0.01
MDA	0.265	0.05	0.256	0.002	0.249	0.001
TAC	-0.368	0.001	-0.238	0.002	-0.456	0.001
Mineral elements	0.358	0.001	0.367	0.004	0.357	0.01
WISC- IQ score	0.325	0.002	0.213	0.01	0.256	0.001

**Fig. 2:**
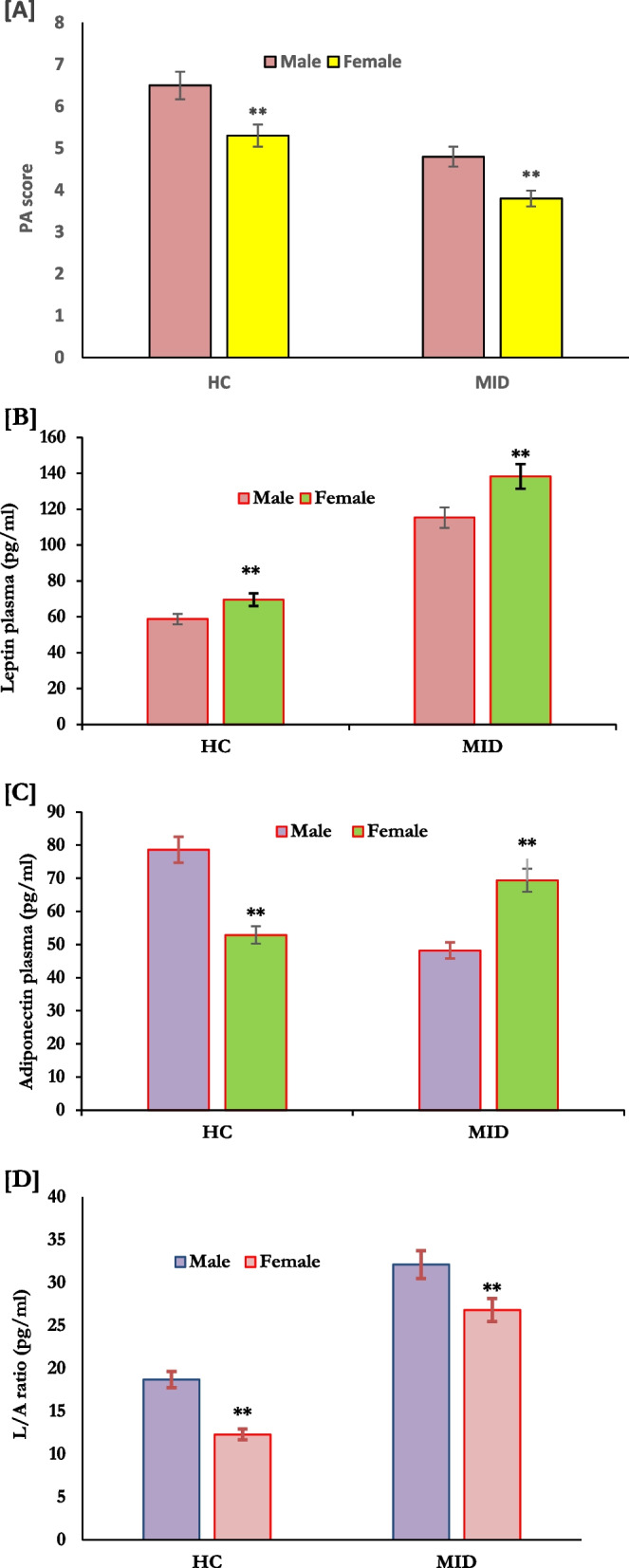
Effect of Gender on physical activity (**A**), plasma levels of adipokines (pg/mL); leptin (**B**), Adiponectin (**C**), and L/A ratio (**D**) in HC (*n* = 180) and adloscence withMID (*n* = 120). HC: healthy controls, MID: moderate intellectual diability. **p < 0.01. ***p < 0.001 ManneWhitney test

**Fig. 3:**
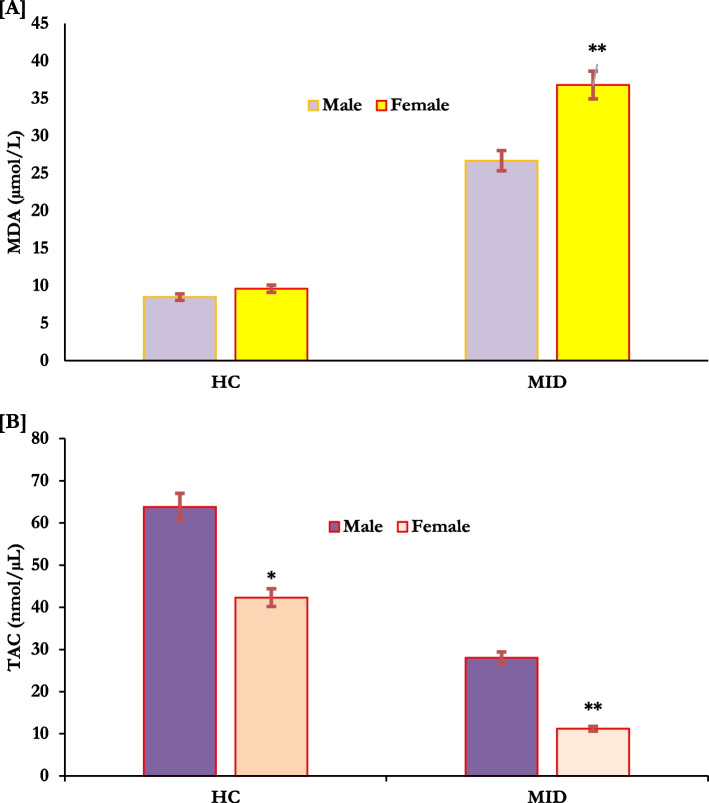
Effect of Gender on oxidative stress plasma levels MDA (**A**) and TAC (**B**) in HC (*n* = 180) and adloscence withMID (*n* = 120). *p < 0.01. **p < 0.001 Manne Whitney test. MDA: malondialdehyde, TAC: total antioxidant capcity, HC: healthy controls, MID: moderate intellectual diability

Regarding gender effect on physical activity, adipokines levels, and oxidative stress, girls with MID had lower physical activity scores than males in the same group (Fig. 2A). Also, higher leptin and L/A ratios with lower plasma adiponectin levels were reported in girls with MID compared to males of the same group (Fig. 2B, C and D). However, in normal control subjects, there were comparable levels of the studied parameters; leptin (*p* = 0.001), adiponectin (*p* = 0.001), and L/A ratio (*p* = 0.001) in boys compared to healthy girls as shown in Fig. 2B, C and D). In addition, a significant increase in the levels of MDA and a decrease in the levels of TAC activity were reported in girls (*P* = 0.001) compared to men of the same group ( Fig. 3A and B).

## Discussion

Intellectual and developmental disabilities are broadly conceptualized to include a range of physical, mental, and behavioral impairments [26]. Patients with intellectual disabilities have been shown to have a limited capacity for cooperation, communication, and other biological consequences, which significantly requires a specialized interest from healthcare professionals worldwide [50, 51].

In this study, intellectual disability of moderate type (MID) was reported in 40% of the studied populations, most of whom are men aged 12–18 years (66.6% for men vs. 33.3 for females). The incidence of MID among the studied sample was linked with the release of adiposity markers; BMI, WHR, and WHtR, and lower physical activity compared to healthy control subjects. Matched with our results, obesity was significantly reported in children or adolescents with intellectual disabilities (ID); it was nearly twice the prevalence for those without ID (28.9% vs. 15.5%) [23, 24]. This may be related to poor physical activity and longer time spent sitting in front of screen-based media [26–30].

Supported data also recently reported that childhood overweight or obesity is clearly very pervasive or problematic among healthy children and those with ID [23, 52–55]. This is commonly attributed to poor physical activity and increased sedentary lifestyles, such as excess food intake and screen-based media use for longer periods [23, 52–55]. In addition, an elevated weight status among youth with ID is the leading risk for mental health problems and increased morbidity and mortality rates among adults with ID [55]. Thus, increasing physical activity and a self-monitoring diet were recommended among younger and older ages to yield clinically meaningful weight losses among adults with ID, reducing the severity of ID-related consequences [26, 56, 57].

Despite the prevalence of obesity increasing and prevailing among people with disabilities [58, 59], surprisingly, no or little attention has been paid to addressing the profile or potential roles of adipokines as measures of adiposity and metabolic disorders and oxidative stress (OS) among youth with disabilities [60].

In the present study, adiponectin and leptin, the most essential adipokines associated with adiposity and metabolic disorders, were estimated among adolescents e with ID disabilities. Changes in plasma levels of leptin, L/A ratio, and adiponectin are significantly associated with the incidence of ID among adolescents. Higher plasma levels of leptin and L/A ratio and lower adiponectin concentrations were significantly reported in adolescents with MID (*P* = 0.001) compared with the healthy controls.

In addition, MDA and TAC as parameters of oxidative stress were significantly evaluated in this study. The levels of MDA significantly increased, and TAC significantly decreased in adolescents with MID (*P* = 0.001) compared to the healthy controls.

In adolescents, obesity was associated with severe health complications such as mental disorders, long-term vascular complications, oxidative stress, and higher rates of severe metabolic syndrome [61, 62] previously. At younger ages, the lower levels of adiponectin and the higher levels of leptin were shown to be associated with the risk for mental health problems, particularly ID [26, 23, 52–62]. Similarly, the levels of OS measured by MDA were significantly higher, along with a reduction in TAC activity in persons with ID compared to the control group [38, 39, 46].

In this current study, leptin, adiponectin, and L/A were measured as markers of adiposity in subjects with ID correlated negatively with BMI, WHtR, PA scores, and TAC and positively with gender, WISC-IQ score, and MDA. A cascade of events characterized by an asymptomatic inflammatory process, including inflammatory cytokines along with oxidative stress significantly associated with the severity of intellectual disabilities (ID) among older and younger ages [46, 62, 63]. Thus, monitoring the levels of oxidative and adipokine molecules could serve as biomarkers of ID which may allow early diagnosis and intervention and improve the quality of care for persons with ID.

In obese people, the metabolic disturbances are decompensated. Although overweight is a preclinical condition, obesity is a clinically manifested metabolic disorder, including mineral imbalances [12], which could play a potential role in the pathogenesis of intellectual disabilities (ID).

In this study, plasma mineral elements were estimated in all participants. A significant increase in the levels of Fe, Mn, Zn, Hg, Pb, Ca, Cr, Mg, and Ni, and a decrease in the levels of Al, Na, K, Cu, and Zn/Cu ratio were reported in cases with MID compared to healthy controls. Changes in the levels of mineral elements correlated positively with plasma levels of adipokines; leptin, adiponectin, L/A ratio, MDA, TAC, and ID score (WISC-IQ score) and negatively with adiposity parameters; BMI, WHR, and WHtR. In addition, the increment of Fe, Mn, Zn, Hg, Pb, Ca, Cr, Mg, Ni, and decrement in the levels of Al, Na, K, Cu, and Zn/Cu ratio showed no significant effect with gender in subjects with ID.

Mineral elements as essential nutrients showed potential regulatory, immunologic, and antioxidant functions in biological systems [10]. Their potential action was significantly related to their incorporation as essential components or cofactors of enzymes throughout cellular metabolism [10]. Thus, it was reported that trace elements and minerals interfere with the pathogenesis of obesity and its complications, such as mental diseases, mainly through their involvement in the processes of peroxidation and inflammation [11]. Reduced IQ and cognitive functions, learning difficulties, and impaired growth were reported in children with Pb blood levels below 10 μg·dL − 1 [34–37]. Also, the pathophysiology of mental intoxication and producing intellectual or developmental defects may proceed via cellular free radical oxidative stress mechanisms [38, 39]. In recent studies, the levels of iron, copper, and zinc were lower in the plasma / serum of the children with intellectual disabilities compared to typically developing controls [64–68]. However, the relationship between physiological antagonists and intellectual activity is less clear. While some studies have suggested that excess intake of certain minerals can interfere with the absorption or utilization of other essential minerals [69–71], it is not clear how this affects cognitive function, and intellectual abilities in children and adolescents.

The usual normal human health needs adequate amounts of essential and trace elements with optimum levels either increasing or decreasing according to the vital cellular processes [72–76]. It was reviewed previously that the administration of selective antioxidants along with essential trace elements and minerals were required efficiently to reduce the extent of oxidative damage and related complications and to avoid serious diseases such as beta-thalassemia major and other brain-related disorders [76]. Elements and minerals should be present in the body in appropriate amounts and must be available for reacting with other elements to form critical molecules as well as to participate in various important chemical reactions [77].

According to the effect of gender, a clinical change in the levels of adipokines; leptin, adiponectin, L/A-ratio, oxidative stress; MDA, TAC, and detrimental changes in the levels of essential trace elements were reported in girls with MID compared to men of the same category.

In addition to that, the levels of adipokines and trace elements were clinically associated with adiposity parameters; BMI, WHR, WHtR, and the severity score (WISC-IQ score) of the severity of intellectual disabilities (ID). It was suggested previously that the inadequate ingress of trace elements into the biological cells may provide deleterious effects on different tissue functions and may lead to disease [78]. For this reason, analyzing changes to oligo-element concentrations in patients with MID could lead to a better understanding of any functional abnormalities associated with MID [78–80].

Finally, significant changes in plasma concentrations of plasma mineral elements were reported in obese adolescents with MID, which correlated positively with oxidative stress parameters; MDA, TAC, and adipokines; leptin, adiponectin, L/A ratios, and other related biomarkers of adiposity.

### Strengthen and limitations

Our study had several limitations. Although our study generally showed the importance of identifying the levels of mineral elements and their association with obesity and intellectual disability scores among younger aged 12–18 individuals, the lack of association between compromised nutritional status due to factors such as feeding difficulties, limited food choices, and medication side effects should be addressed to evaluate long-lasting changes of mineral elements and their essential roles in the pathogenesis of intellectual disability among younger ages. Our results can be interpreted as preliminary findings. Thus, further studies based on long follow-ups are recommended to understand the potential association of mineral elements with intellectual activities.Therefore, individualized assessments of nutritional status and mineral intake are important for guiding appropriate interventions and monitoring the progress of intellectual abilities among children and adolescents. In addition, our study recommended that it is important for students with moderate intellectual disabilities to receive adequate levels of essential minerals in their diet to support their overall health and well-being. A balanced and varied diet that includes a variety of nutrient-dense foods can help ensure adequate mineral intake.

## Conclusions

Moderate intellectual disability (MID) was associated with abnormal changes in essential mineral elements and adipokines levels and increased levels of cellular oxidative stress. Thus, evaluation of the plasma mineral and trace elements, and adipokines levels is used as a potential diagnostic parameter in diagnosing MID.

## Data Availability

All data generated or analyzed during this study are presented in the manuscript. Please contact the corresponding author for access to the data presented in this study.
